# 5-2-1 Criteria: A Simple Screening Tool for Identifying Advanced PD Patients Who Need an Optimization of Parkinson's Treatment

**DOI:** 10.1155/2020/7537924

**Published:** 2020-03-24

**Authors:** D. Santos-García, T. de Deus Fonticoba, E. Suárez Castro, A. Aneiros Díaz, D. McAfee

**Affiliations:** ^1^Complejo Hospitalario Universitario de A Coruña (CHUAC), A Coruña, Spain; ^2^Complejo Hospitalario Universitario de Ferrol (CHUF), Ferrol, A Coruña, Spain; ^3^University of Pennsylvania, Philadelphia, PA, USA

## Abstract

**Objective:**

5- (5 times oral levodopa tablet taken/day) 2- (2 hours of OFF time/day) 1- (1 hour/day of troublesome dyskinesia) criteria have been proposed by a Delphi expert consensus panel for diagnosing advanced Parkinson's disease (PD). The aim of the present study is to compare quality of life (QoL) in PD patients with “5-2-1 positive criteria” vs QoL in PD patients without “5-2-1 positive criteria” (defined as meeting ≥1 of the criteria).

**Methods:**

This is a cross-sectional, observational, monocenter study. Three different instruments were used to assess QoL: the 39-Item Parkinson's Disease Quality of Life Questionnaire Summary Index Score (PDQ-39SI); a subjective rating of perceived QoL (PQ-10); and the EUROHIS-QOL 8-Item Index (EUROHIS-QOL8).

**Results:**

From a cohort of 102 PD patients (65.4 ± 8.2 years old, 53.9% males; disease duration 4.7 ± 4.5 years), 20 (19.6%) presented positive 5-2-1 criteria: 6.9% for 5, 17.6% for 2, and 4.9% for 1. 37.5% (12/32) and 25% (5/20) of patients with motor complications and dyskinesia, respectively, presented 5-2-1 negative criteria. Both health-related (PDQ-39SI, 25.6 ± 14 vs 12.1 ± 9.2; *p* < 0.0001) and global QoL (PQ-10, 6.1 ± 2 vs 7.1 ± 1.3; *p*=0.007; EUROHIS-QOL8, 3.5 ± 0.5 vs 3.7 ± 0.4; *p*=0.034) were worse in patients with 5-2-1 positive criteria. Moreover, nonmotor symptoms burden (Non-Motor Symptoms Scale total score, 64.8 ± 44.8 vs 39.4 ± 35.1; *p* < 0.0001) and autonomy for activities of daily living (ADLS scale, 73.5 ± 13.1 vs 89.2 ± 9.3; *p* < 0.0001) were worse in patients with 5-2-1 positive criteria. Patient's principal caregiver's strain (Caregiver Stain Index, 4.3 ± 3 vs 1.5 ± 1.6; *p* < 0.0001), burden (Zarit Caregiver Burden Inventory, 28.4 ± 12.5 vs 10.9 ± 9.8; *p* < 0.0001), and mood (Beck Depression Inventory II, 12.2 ± 7.2 vs 6.2 ± 6.1; *p* < 0.0001) were worse in patients with 5-2-1 positive criteria as well.

**Conclusions:**

QoL is worse in patients meeting ≥1 of the 5-2-1 criteria. This group of patients and their caregivers are more affected as a whole. These criteria could be useful for identifying patients in which it is necessary to optimize Parkinson's treatment.

## 1. Introduction

In Parkinson's disease (PD), effective management is key at all stages and often requires individual customization of therapy as the disease progresses [1]. However, in the absence of a biomarker, a diagnostic test, or a gold standard index to determine the severity of PD (based on the motor and nonmotor symptoms), clinicians often rely on varied clinical evaluation and medical history to determine staging in PD [2]. Recently published systematic reviews and consensus articles acknowledge the growing need to establish guidelines for the different treatment approaches for advanced PD patients [3–8]. At this stage, it is important to ensure timely referral of patients to a movement disorder specialist before deterioration of quality of life (QoL) and development of complications of advancing disease [9, 10]. Previous studies have observed that motor fluctuations are frequent even during the first years after the diagnosis of PD. Motor fluctuations also are related to a greater NMS burden, a worse QoL, and less functional independence for activities of daily living [11, 12]. Having an easy tool to identify which patients are worse is necessary for clinical practice because the adjustment of symptomatic treatment will improve the patients' quality of life and autonomy.

In this context, 5- (5 times oral levodopa tablet taken/day) 2- (2 hours of OFF time/day) 1 (1 hour/day of troublesome dyskinesia) criteria have recently been proposed by a Delphi expert consensus panel for diagnosing advanced PD [8, 13]. However, these criteria have been only applied in one cohort of advanced PD patients under levodopa infusion therapy (ADEQUA Study) [14]. The objective of the present study was to compare functional dependency in PD patients with vs without “5-2-1 positive criteria” (defined as meeting ≥1 of the criteria). Moreover, nonmotor symptoms (NMS) burden, patients' QoL, and the patient's principal caregiver status was also compared between both groups to determine if 5-2-1 criteria could be a useful screening tool for identifying advanced PD patients who need an optimization of Parkinson's treatment.

## 2. Methods

A subgroup of PD patients from the COPPADIS cohort-2015 were included in this study. Methodology about the COPPADIS-2015 study has been previously published [15]. This is a multicenter, observational, longitudinal-prospective, 5-year follow-up study that was designed to analyze disease progression in a Spanish population of PD patients. The data for the present study (cross-sectional study) were obtained from the baseline evaluation of PD patients assessed at one center (CHUF, Ferrol, Spain), that was from January 2016 to October 2017. All patients included were diagnosed according to UK PD Brain Bank criteria. Exclusion criteria were: non-PD parkinsonism, dementia, age <18 or >75 years, inability to read or understand the questionnaires, to be receiving any advanced therapy (continuous infusion of levodopa or apomorphine, and/or with deep brain stimulation), and presence of comorbidity, sequelae, or any disorder that could interfere with the assessment [15].

Information on sociodemographic aspects, factors related to PD, comorbidity, and treatment was collected. Patient baseline evaluation included motor assessment (H&Y, Unified Parkinson's Disease Rating Scale [UPDRS] part III and part IV, and Freezing of Gait Questionnaire [FOGQ]), nonmotor symptoms (Non-Motor Symptoms Scale (NMSS), Parkinson's Disease Sleep Scale (PDSS), Visual Analog Scale-Pain (VAS-Pain), and Visual Analog Fatigue Scale (VAFS)), cognition (Parkinson's Disease Cognitive Rating Scale (PD-CRS) and completing a simple 16-piece puzzle), mood and neuropsychiatric symptoms (Beck Depression Inventory-II (BDI-II), Neuropsychiatric Inventory (NPI), and Questionnaire for Impulsive-Compulsive Disorders in Parkinson's Disease-Rating Scale (QUIP-RS)), disability (Schwab and England Activities of Daily Living Scale (ADLS])), health-related QoL (the 39-Item Parkinson's Disease Questionnaire (PDQ-39SI)), and global QoL (PQ-10 and EUROHIS-QOL 8-Item Index (EUROHIS-QOL8)). In patients with motor fluctuations, the motor assessment was conducted during the OFF state (without medication in the last 12 hours) and during the ON state. However, in patients without motor fluctuations, the assessment was only performed without medication (first thing in the morning without taking medication in the previous 12 hours). Strain and burden (Caregiver Strain Index (CSI) and Zarit Caregiver Burden Inventory (ZCBI)), mood (BDI-II), and QoL (PQ-10 and EUROHIS-QOL8) were also assessed in the patient's principal caregiver.

The information about the criteria of the 5-2-1 concept (frequency of daily levodopa tablet intake, OFF time, and dyskinesia time and severity of dyskinesia) had been collected at baseline evaluation after UPDRS-IV application and asked directly to the patients. A patient with 5-2-1 positive criteria was considered when at least 1 of the 3 criteria was positive [13].

Considering functional independency for activities of daily living as the gold standard, the 5-2-1 criteria were applied with the aim of knowing sensitivity and specificity of 5-2-1 criteria for detection of functional dependency. Functional dependency was defined to be present if the ADLS score was less than 80% (80% = completely independent in most chores; 70% = not completely independent) [16].

### 2.1. Data Analysis

Data were processed using SPSS 20.0 for Windows. For comparisons between patients with and without 5-2-1 positive criteria, Student's *t*-test, Mann–Whitney *U* test, chi-square test, or Fisher test, as appropriate, were used (distribution for variables was verified by the one-sample Kolmogorov–Smirnov test). The *p* value was considered significant when it was <0.05.

### 2.2. Standard Protocol Approvals, Registrations, and Patient Consents

For this study, we received approval from the appropriate local and national ethical standards committee. Written informed consents from all participants participating in this study were obtained before the start of the study. COPPADIS-2015 was classified by the AEMPS (*Agencia Española del Medicamento y Productos Sanitarios*) as a Postauthorization Prospective Follow-Up study with the code COH-PAK-2014-01.

## 3. Results

Out of 102 PD patients included (65.4 ± 8.2 years old, 53.9% males; disease duration 4.7 ± 4.5 years), 20 (19.6%) presented positive 5-2-1 criteria: 6.9% for 5, 17.6% for 2, and 4.9% for 1. 37.5% (12/32) and 25% (5/20) of patients with motor complications and dyskinesia, respectively, presented 5-2-1 negative criteria. Patients with 5-2-1 positive criteria presented longer disease duration and a worse motor status and were receiving a higher equivalent levodopa daily dose (Table 1). NMS burden was significantly greater in patients with 5-2-1 positive criteria (NMSS total score, 64.8 ± 44.8 vs 39.4 ± 35.1; *p* < 0.0001) as the score of domains 2 (sleep/fatigue), 4 (perceptual problems), and 9 (miscellaneous) were all greater in this group. Severe to very severe NMS burden (NMSS total score  >40) [17] was more frequent in patients with 5-2-1 positive criteria (60% vs 31.7%; *p*=0.019). Moreover, the observed score on different scales (BDI-II, NPI, PDSS, VAS-Pain, and VAFS) indicated a greater nonmotor affectation in PD patients with 5-2-1 criteria compared to patients with negative criteria (Table 1).

Regarding QoL, both health-related (PDQ-39SI, 25.6 ± 14 vs 12.1 ± 9.2; *p* < 0.0001) and global QoL (PQ-10, 6.1 ± 2 vs 7.1 ± 1.3; *p*=0.007; EUROHIS-QOL8, 3.5 ± 0.5 vs 3.7 ± 0.4; *p*=0.034) were worse in patients with 5-2-1 positive criteria (Table 2). All score domains of the PDQ-39SI except 5 (social support), 6 (cognition), and 7 (communication) were significantly higher in patients with 5-2-1 positive criteria. In the case of global QoL, significant differences between groups were observed for QoL rate and satisfaction with the ability to perform the daily living activities. Interestingly, patients with motor fluctuations and 5-2-1 positive criteria (*n* = 20) tended to have a worse QoL than those with motor fluctuations but negative criteria (*n* = 12) (PDQ-39SI, 25.6 ± 14 vs 19.2 ± 7.3; *p*=0.157). Moreover, functional capacity for activities of daily living was significantly worse in patients with 5-2-1 positive criteria (ADLS, 73.5 ± 13.1 vs 89.2 ± 9.3; *p* < 0.0001). The 5-2-1 criteria presented a sensitivity and specificity of 92.7% and 45%, respectively, for identifying patients with functional dependency (Table 2).

Finally, strain (CSI, 4.3 ± 3 vs 1.5 ± 1.6; *p* <0.0001), burden (ZCBI, 28.4 ± 12.5 vs 10.9 ± 9.8; *p* < 0.0001), and mood (BDI-II, 12.2 ± 7.2 vs 6.2 ± 6.1; *p* < 0.0001) were significantly worse in patient's principal caregiver with 5-2-1 positive criteria compared to those with negative criteria (Table 3). Although there were no differences in QoL between both groups, there was a trend of a worse QoL in patient's principal caregiver with 5-2-1 positive criteria.

## 4. Discussion

The present study applies the 5-2-1 criteria for the first time in a general cohort of PD patients. This study also demonstrates that both health-related and global QoL are worse in those patients with 5-2-1 positive criteria. Moreover, patients' NMS burden was greater and autonomy for activities of daily living was also worse. Patient's principal caregiver strain, burden, and mood were worse in this group of patients as well.

Currently, treatments for PD are symptomatic and the objective is to improve patients' autonomy and QoL [18]. In advanced PD patients, this is an even more necessary goal because QoL in them is worse [19]. Indeed, changes in QoL has been chosen as the principal variable in some studies with PD patients selected for receiving a second line therapy [20, 21] and the use of PDQ-39 in routine care for PD has been suggested [22]. However, although some criteria have been proposed with the aim to define advanced PD [23–25], in many cases the criteria are not useful in daily clinical practice because their application is time-consuming and also because the concept of PD is too broad [26]. Therefore, a quick screening tool that identifies which PD patients are worse and need optimized treatment, including possibly a second line therapy, would be ideal. The 5-2-1 criteria could be a useful screening tool. Firstly, its application is simple and would only need to know the number of medication doses and the time per day in the OFF state with disabling dyskinesia. Secondly, these criteria seem to identify patients with worse QoL and with more requirements to improve their status. Being that patients in this cohort with 5-2-1 positive criteria presented a longer disease duration and they were more affected as a whole, it makes sense that patients with motor complications but with 5-2-1 negative criteria tended to have a better QoL compared to those with positive criteria. So, these criteria could differentiate between patients with more severe motor complications and those with less severe motor complications. In fact, the 5-2-1 criteria resulted in a high negative predictive value for functional dependency since only 7% of the patients were functionally dependent when the criteria were negative. Finally, it is easy to keep in mind the concept of 5-2-1 for application in real clinical practice.Previous studies have analyzed the reasons driving treatment modification in PD [27, 28]. The most common reasons behind the anti-Parkinson drug therapy changes among neurologists were presence/worsening of motor or NMS. Patients attributed greater relevance than neurologists to NMS as a reason requiring treatment changes. In our study, patients with 5-2-1 positive criteria presented more severe motor and NMS. Specifically, mood, neuropsychiatric symptoms, sleep problems, fatigue, and pain were more severe in patients with 5-2-1 positive criteria and the percentage of patients with severe to very severe NMS burden was the double in this group compared to those patients with negative criteria. On the other hand, some studies have shown improvement in the caregiver status of patients with advanced PD after starting some second-line therapies [29, 30]. An overburdened caregiver cares less which contributes to a worse QoL of the patient, so it is important to identify the problem and take action. In our study, 5-2-1 criteria served to identify the most overburdened and most poor-minded caregivers.

The present study has some limitations. This is a cross-sectional study and not a longitudinal study. Motor complications were assessed using the UPDRS-part IV applied by an expert neurologist on PD, but other more sensitive tools exist [31, 32]. The statistical significance in the analysis for the subgroup of PD patients with motor fluctuations but 5-2-1 negative criteria was limited by the size of the sample (few patients). Our sample was not fully representative of the PD population due to inclusion and exclusion criteria (i.e., age limit, no dementia, no severe comorbidities, and no second line therapies). Finally, 5-2-1 criteria should be tested in a larger cohort of PD patients and follow-up after interventions in relation with the results of the test should be assessed.

As conclusion, the first application of 5-2-1 criteria in a cohort of PD patients demonstrates that QoL is worse in patients meeting ≥1 of the 5-2-1 criteria. This group of patients and their caregivers were more severely burdened. These criteria could be useful for identifying patients in which it is necessary to optimize Parkinson's treatment.

## Figures and Tables

**Table 1:** PD-related variables in patients with 5-2-1 criteria (*n* = 20) vs those with 5-2-1 negative criteria (*n* = 82).

	5-2-1 positive (*n* = 20)	5-2-1 negative (*n* = 82)	*p* value
Age	66.2 ± 10.4	65.2 ± 7.6	0.634
Disease duration (years)	11.4 ± 3.9	3.1 ± 2.8	**<0.0001**
Sex (female)	65	41.5	0.058
Hoehn and Yahr scale:			**<0.0001**
Stage 1	0	27.3	
Stage 2	65	68.8	
Stage 3–5	35	3.9	
UPDRS-III	27.9 ± 6.4	16.3 ± 7.5	**< 0.0001**
UPDRS-IV	5.7 ± 2.1	1.2 ± 1.4	**< 0.0001**
FOGQ	7.7 ± 5.4	1.7 ± 3	**< 0.0001**
Daily dose L-dopa (mg)	612.2 ± 230.8	202.7 ± 239.6	**< 0.0001**
Eq. daily dose L-dopa (mg)	826.3 ± 434.4	342.4 ± 318.7	**< 0.0001**
Time under L-dopa (months)	105.2 ± 49.3	18 ± 29.1	**< 0.0001**
PD-CRS	90.7 ± 14.9	92.1 ± 13.4	0.676
NMSS	64.8 ± 44.8	39.4 ± 35.1	**0.007**
Cardiovascular	3.3 ± 6.1	4.4 ± 10	0.642
Sleep/fatigue	22.7 ± 23	14.1 ± 14.4	**0.038**
Mood/apathy	18.2 ± 23.6	10.8 ± 16.4	0.100
Perceptual symptoms	8 ± 15.8	1.8 ± 6.4	**0.006**
Attention/memory	12.2 ± 18.6	7.3 ± 11	0.130
Gastrointestinal symptoms	12.3 ± 15.7	8 ± 14.9	0.247
Urinary symptoms	25.3 ± 28	18.5 ± 21.8	0.242
Sexual dysfunction	22.5 ± 25	24.6 ± 30.4	0.775
Miscellaneous	28.6 ± 18.3	10.7 ± 12.8	**<0.0001**
BDI-II	12.1 ± 10.5	7.3 ± 6.2	**0.010**
NPI-subject	14.1 ± 12	6.4 ± 6.7	**0.002**
QUIP-RS	2.1 ± 5.1	0.8 ± 2.9	0.135
PDSS	116.3 ± 19.4	126.9 ± 14.9	**0.008**
VAS-PAIN	4.5 ± 2.7	2.8 ± 2.6	**0.009**
VASF-physical	4.5 ± 2.7	2.8 ± 2.6	**0.016**
VASF-mental	3.4 ± 2.7	1.9 ± 2.6	**0.024**

Chi-squared and Mann–Whitney–Wilcoxon tests were applied. The results represent percentages or mean ± SD. Data about H&Y and UPDRS-III are during the OFF state (first thing in the morning without taking medication in the previous 12 hours). In comparison between NMSS domains in PD patients, each domain is expressed as a percentage ([score/total score] × 100). ADLS, Schwab and England Activities of Daily Living Scale; BDI-II, Beck Depression Inventory-II; FOGQ, Freezing of Gait Questionnaire; NMSS, Non-Motor Symptoms Scale; NPI, Neuropsychiatric Inventory; PD, Parkinson's disease; PD-CRS, Parkinson's Disease Cognitive Rating Scale; PDQ-39SI, 39-Item Parkinson's Disease Quality of Life Questionnaire Summary Index; PDSS, Parkinson's Disease Sleep Scale; QUIP-RS, Questionnaire for Impulsive-Compulsive Disorders in Parkinson's Disease-Rating Scale; UPDRS, Unified Parkinson's Disease Rating Scale; VAFS, Visual Analog Fatigue Scale; VAS-Pain, Visual Analog Scale-Pain.

**Table 2:** QoL and autonomy for activities of daily living in patients with 5-2-1 criteria (*n* = 20) vs those with 5-2-1 negative criteria (*n* = 82).

	5-2-1 positive (*n* = 20)	5-2-1 negative (*n* = 82)	*p* value
PDQ-39SI	25.6 ± 14	12.1 ± 9.2	**<0.0001**
Mobility	32.1 ± 22.4	11.7 ± 17.7	**<0.0001**
Activities of daily living	25.8 ± 15.3	12.1 ± 12	**<0.0001**
Emotional well-being	29.1 ± 27.5	16.1 ± 18.9	**0.014**
Stigma	16.8 ± 32.4	6 ± 14.9	**0.028**
Social support	7.1 ± 16.5	2.5 ± 9.3	0.102
Cognition	22.2 ± 21	14.2 ± 15.8	0.060
Communication	2.9 ± 7.8	2.7 ± 7.7	0.927
Pain and discomfort	52.4 ± 26.6	29.8 ± 20.4	**<0.0001**
PQ-10	6.1 ± 2	7.1 ± 1.3	**0.007**
EUROHIS-QOL8	3.5 ± 0.5	3.7 ± 0.4	**0.034**
Quality of life	3.4 ± 0.9	3.8 ± 0.6	**0.024**
Health status	3 ± 0.8	3.3 ± 0.7	0.069
Energy	3.6 ± 0.6	3.7 ± 0.6	0.452
Autonomy for ADL	2.9 ± 0.7	3.6 ± 0.6	**<0.0001**
Self-esteem	3.6 ± 1.2	3.8 ± 0.7	0.313
Social relationships	3.9 ± 0.8	4 ± 0.5	0.657
Economic capacity	3.8 ± 0.6	3.8 ± 0.6	0.968
Habitat	4 ± 0.8	4.1 ± 0.5	0.452
ADLS	73.5 ± 13.1	89.2 ± 9.3	**<0.0001**
Functional dependency (%)	45	7.3	**<0.0001**

Chi-squared and Mann–Whitney–Wilcoxon tests were applied. ADLS, Schwab and England Activities of Daily Living Scale; PDQ-39SI, 39-Item Parkinson's Disease Quality of Life Questionnaire Summary Index.

**Table 3:** Strain, burden, mood, and QoL in patient's principal caregiver of those patients with 5-2-1 criteria (*n* = 16) vs those with 5-2-1 negative criteria (*n* = 46).

	5-2-1 positive (*n* = 16)	5-2-1 negative (*n* = 46)	*p* value
CSI	4.3 ± 3	1.5 ± 1.6	**<0.0001**
High stress level (≥7)	31.2	0	**0.001**
ZCBI	28.4 ± 12.5	10.9 ± 9.8	**<0.0001**
Little or no burden (0–20)	25	82.6	**<0.0001**
Mild to moderate burden (21–40)	62.5	17.4	
Moderate to severe burden (41–60)	12.5	0	
Severe burden (61–88)	0	0	
BDI-II	12.2 ± 7.2	6.2 ± 6.1	**<0.0001**
PQ-10	6.4 ± 1.5	7.1 ± 1.6	0.141
EUROHIS-QOL8	3.6 ± 0.5	3.8 ± 0.5	0.139
Quality of life	3.5 ± 0.6	3.8 ± 0.8	0.186
Health status	3.6 ± 0.6	3.6 ± 0.9	0.908
Energy	3.5 ± 0.7	3.8 ± 0.8	0.253
Autonomy for ADL	3.6 ± 0.8	3.8 ± 0.8	0.495
Self-esteem	3.4 ± 0.6	3.8 ± 0.7	0.057
Social relationships	3.7 ± 0.8	3.9 ± 0.6	0.252
Economic capacity	3.6 ± 0.9	3.9 ± 0.7	0.132
Habitat	4 ± 0.5	4.2 ± 0.6	0.283

Chi-squared and Mann–Whitney–Wilcoxon tests were applied. The results represent percentages or mean ± SD. ZCBI and CSI were classified in groups according to severity [27]. BDI-II, Beck Depression Inventory-II; CSI, Caregiver Strain Index; ZCBI, Zarit Caregiver Burden Inventoy.

## Data Availability

The data used to support the findings of this study are available from the corresponding author upon request.

## Abbreviations

ADLS: Schwab and England Activities of Daily Living Scale
BDI: Beck Depression Inventory-II
FOGQ: Freezing of Gait Questionnaire
NMS: Nonmotor symptoms
NMSS: Non-Motor Symptoms Scale
NPI: Neuropsychiatric Inventory
PD: Parkinson's disease
PD-CRS: Parkinson's Disease Cognitive Rating Scale
PDQ-39SI: 39-Item Parkinson's Disease Quality of Life Questionnaire Summary Index
PDSS: Parkinson's Disease Sleep Scale
QUIP-RS: Questionnaire for Impulsive-Compulsive Disorders in Parkinson's Disease-Rating Scale
UPDRS: Unified Parkinson's Disease Rating Scale
VAFS: Visual Analog Fatigue Scale
VAS-Pain: Visual Analog Scale-Pain.
